# Forging Prawn and Salmon Flavours with Non-Animal-Based Ingredients

**DOI:** 10.3390/foods14050820

**Published:** 2025-02-27

**Authors:** Jiaqiang Luo, Damian Frank, Jayashree Arcot

**Affiliations:** 1 Food and Health Group, School of Chemical Engineering, Faculty of Engineering, UNSW Sydney, Kensington, NSW 2052, Australia; j.arcot@unsw.edu.au; 2 All G, Waterloo, NSW 2017, Australia; damian@allg.com

**Keywords:** alternative protein, flavour, formulation, volatile, odour

## Abstract

The development of plant-based seafood alternatives with authentic flavour profiles remains a significant challenge, limiting their appeal to seafood consumers. This study hypothesised that incorporation of flavour precursors including free amino acids, betaine, and long chain omega-3 fatty acids would enhance the flavour resemblance of plant-based prawn and salmon prototypes to their authentic seafood counterparts. Prototypes were analysed using headspace solid-phase microextraction gas chromatography–mass spectrometry and evaluated by a semi-trained sensory panel. Volatile analysis revealed 64 compounds across prototypes, with significant variations attributed to precursor combinations and thermal treatments. Frying enhanced volatile profiles, particularly in plant-based prawn prototypes fortified with all three flavour precursors, producing key prawn odourants, including pyrazines and trimethylamine. Notably, betaine pyrolysis under moderate cooking conditions was demonstrated as a potential pathway for trimethylamine formation, contributing to fish-like odours. Sensory evaluation showed that the final plant-based prawn prototype exhibited strong cooked crustacean and grilled notes, aligning with the observed volatile profile. While the salmon prototype displayed key salmon odourants, its cooked salmon odour was less pronounced, suggesting a need for a more robust flavouring strategy. This study highlights the potential of targeted flavour precursor formulations to improve the flavour quality of plant-based seafood alternatives, paving the way for their wider acceptance.

## 1. Introduction

In the global plant-based (PB) protein market, the PB seafood alternative (PBSA) sector remains relatively small compared to meat and dairy analogues. However, despite its global market size being valued at only USD 42.1 million in 2021, it is projected to reach USD 1.3 billion by 2031, making it the fastest-growing sector within the PB protein alternative industry [1]. Despite this optimistic forecast, both person- and product-related barriers inhibit the wider acceptance of PBSAs. Factors such as flavour, dietary preferences, product pricing, product availability, and naturalness all play a role [2]. Amongst these barriers, sensory qualities, particularly flavour, are fundamental to product acceptability and largely depend on the use of flavouring agents, whether chemically synthesised or derived from natural sources.

The first step in an effective flavouring process for PBSAs is to gain a thorough understanding of the flavour profile of the target seafood. Numerous studies have been conducted to profile inherent flavour molecules and precursor compounds, both of which play a crucial role in shaping the flavour profile of cooked seafood. Due to their high abundance and contribution to umami and sweetness [3], free amino acids (FAAs) together with 5′-nucleotides significantly influence seafood taste profiles [4,5,6]. In addition to directly contributing to taste, FAAs also serve as precursors to seafood odour formation, especially through thermal processing. Strecker degradation of FAAs generates a range of aldehydes that are important for the seafood flavour profile [7]. For instance, methional, derived from methionine, is a common odourant found in cooked crustaceans, molluscs, and finfish [8]. 2-Acetyl-1-pyrroline, a well-established key odourant of cooked prawns [9,10], can be generated during thermal food processing through the Maillard reaction between proline or ornithine and carbohydrates [11]. Furthermore, trimethylamine oxide (TMAO) and betaine (*N*,*N*,*N*-trimethyl glycine) are key osmolyte molecules in seafood species—present at relatively high concentration—that protect cells from osmotic stress, which may contribute to sweet and umami tastes [12]. Both TMAO and betaine can be degraded to trimethylamine (TMA) through microbial activity [13,14], or for betaine, potentially through thermal degradation during cooking. While a high concentration of TMA is considered a biomarker of seafood spoilage, a low concentration of TMA contributes to the fresh fish-like odour, which is essential for shaping fundamental seafood flavour [15]. The thermal degradation of omega-3 polyunsaturated fatty acids (n-3 PUFAs) produces a range of aldehydes and methyl ketones, followed by acids, hydrocarbons, lactones, and alcohols [7]. These compounds contribute to the characteristic volatile profile of cooked oily fish, such as salmon [6,16].

Even with this information available, the formulation of seafood flavour is still far from straightforward, and the flavour quality of commercial PBSAs currently does not live up to expectations of regular seafood consumers and is often disappointing. Recent research has shown that most PBSAs available in the Australian market fail to replicate the flavours of their seafood counterparts, with the exception of PB sashimi products, where flavour plays a relatively minor role [17]. This may be due to the lack of ideal PB seafood flavouring agents and/or flavouring strategies that can accurately introduce the complex and subtle flavour attributes to the PB substrate [17].

Seafood typically has a very mild odour when raw, but its flavour profile changes significantly upon cooking. In contrast, in the production of commercial PBSAs, flavouring agents are generally pre-added during the structuring process and homogenised into the food matrix [18]. However, manufacturers often add excessive amounts of flavouring agents at this stage, one of the important reasons being to mask undesirable flavours like beany odour and bitterness from plant ingredients [19]. This results in overly pronounced odours even before the products are cooked, giving the products an unnatural flavour impression [17]. Upon heating, more complex interactions occur, further shifting the flavour profile away from the intended target.

Numerous studies, including many review articles [20,21,22], have explored potential solutions for overcoming flavour challenges in PB products. However, much of this research has focused on removing undesirable flavours and exploring new PB ingredients and processing methods. Little emphasis has been placed on how ideal PBSA flavouring agents should be formulated to more closely replicate seafood flavour. Our recent investigation into commercial PBSAs revealed that excessively high levels of particular volatile compounds and a lack of signature seafood odourants in these products are common issues that warrant greater attention in flavour formulation. In this study, we explore a formulation strategy based on using flavour precursors. Prawn and salmon were selected as target species due to their widespread popularity worldwide. Besides, prawn is low in fat (~0.9%) and has a relatively high abundance of flavour precursors such as FAAs and betaine, while salmon is high in fat (~19.8%) and rich in n-3 PUFAs, making them ideal targets for testing this flavouring strategy. Using a gel system consisting of hydrocolloids, starch, faba protein isolate, and canola oil, FAAs, betaine, and encapsulated algal DHA were added at different levels to create PB prawn and salmon prototypes. Additionally, the potential formation of TMA through the thermal degradation of betaine under moderate cooking conditions was also investigated, evaluating its potential for inducing fish-like odour in PBSAs. Headspace solid-phase microextraction coupled with gas chromatography–mass spectrometry (HS-SPME-GC–MS) was used to analyse the volatile compositions. Analyses were performed on both thermoset gels and fried thermoset gels, enabling a comprehensive comparison of volatile profiles of different formulations and changes upon cooking. Olfactory evaluation was conducted to assess the odour qualities of the PB prawn and salmon prototypes perceived by a semi-trained panel.

## 2. Materials and Methods

### 2.1. Chemicals

Food-grade FAAs including alanine, arginine, aspartic acid, glutamic acid, glycine, histidine, leucine, lysine, methionine, proline, valine, cystine, cysteine, phenylalanine, and betaine (*N*,*N*,*N*-trimethylglycine) were sourced from various sports nutrition suppliers in Australia. A standard alkane standard mix (C_8_–C_20_) was purchased from Merck (Castile Hill, NSW, Australia). Purified water was obtained from a Milli-Q system (Millipore Australia, Bayswater, VIC, Australia).

### 2.2. Materials

Australian faba protein powder (82.4% protein, Wellness Road, Coles), sunflower oil, deodorised coconut oil, and tapioca flour were purchased from local supermarkets in Sydney. A hydrocolloid mixture was added to facilitate gelling. Microencapsulated algal oil containing a minimum of 10% docosahexaenoic acid (DHA) was supplied by DSM-Firmenich (life’s DHA^TM^ S10-P200).

### 2.3. Prototype Formulation

Macronutrient targets for the prawn and salmon prototypes were determined using records from the Australian Food Composition Database (available at: https://www.foodstandards.gov.au/science-data/monitoringnutrients/afcd, accessed on 12 December 2024). FAA targets for prawn were adapted from the studies of Vázquez-Ortiz, Caire [23] and Zhang, Chen [24] and then converted into salmon targets based on relative ratios determined in our previous study [8]. Similarly, betaine targets for both prawn and salmon were calculated using literature values from squid [25]. Formulation targets for prawn and salmon prototypes are summarised in Table 1. Prawn flavour mix (PFM) and salmon flavour mix (SFM) were prepared by mixing the listed concentrations of FAAs and betaine. In the formulation, protein, saturated fat, unsaturated fat, and omega-3 oil (algal DHA oil) targets were matched with faba protein isolate, coconut oil, sunflower oil, and encapsulated algal oil, respectively. To develop stable gel structures that could withstand the cooking process, the proportions of protein, fats, and water were slightly adjusted from the targets, with hydrocolloid gelling agents and CaCl_2_ added. A total of six prototypes were formulated, including (1) prawn matrix (P, control of the prawn prototype group), (2) prawn matrix + encapsulated algal DHA oil (P + DHA), (3) prawn matrix + encapsulated algal DHA oil + prawn flavour mix (P + DHA + PFM), (4) salmon matrix (S, control of the salmon prototype group), (5) salmon matrix + encapsulated DHA algal oil (S + DHA), and (6) salmon matrix + encapsulated DHA algal oil + salmon flavour mix (S + DHA + SFM). Ingredients of each prototype were mixed using a commercial stand mixer (KitchenAid, MI, USA). The recipes for the prototypes are summarised in Table 2. All prototypes were prepared in triplicate.

### 2.4. Thermal Treatment

An in-house proprietary blend of hydrocolloids that form heat-set thermo-irreversible gels was used. The ingredient mixtures for the prototypes were shaped into uniform disk shapes using silicon moulds (3 cm in diameter and 1.5 cm in height). Samples at this uncooked status were defined as raw (R). The samples were then vacuum-sealed in plastic bags and then heated in a steam oven at 90 °C for 60 min (Unox Cheftop, Unox, Cadoneghe, Italy). After cooling in an ice-water bath, thermally stabilised (TS) samples were formed. TS samples were then subsequently fried at 200 °C for 3 min on each side in a preheated frying pan lightly greased with canola oil spray to create fried thermally stabilised (FTS) samples. The odour qualities of the R, TS, and FTS samples were assessed and recorded by two experienced flavour chemists, with the descriptors provided in Table 2.

### 2.5. Thermal Generation of Trimethylamine from Betaine

To investigate the potential of betaine to form TMA under moderate cooking conditions (at 200 °C), six model reaction mixes were prepared as follows: (1) betaine only, (2) betaine with oils (coconut oil and sunflower oil), (3) betaine with oils and encapsulated algal DHA oil, (4) betaine with an FAA mix, (5) betaine with an FAA mix and oils, (6) betaine with an FAA mix, oils, and encapsulated algal DHA oil. The ingredient quantities were based on those used in preparing prawn prototypes (ingredients of each model reaction mix are provided in Supplementary Table S1). Samples were placed in 20 mL amber headspace vials and sealed with aluminium foil. After baking at 200 °C for 10 min, aluminium foil was replaced with headspace vial fitted caps after baking. The pyrolysis samples were prepared in duplicate.

### 2.6. Volatile Analysis

Volatile compounds were analysed by a GC–MS system (GC–MS-QP-2020 NX, Shimadzu, Kyoto, Japan) coupled to an AOC-6000 autosampler. Volatile extraction was carried out using a 1.10 mm 120 µm DVB/Carbon WR/PDMS SPME arrow fibre. The fibre was exposed to the headspace of 20 mL amber headspace vials of pyrolysis of betaine experiment or containing 3 g of homogenised prototype samples at 40 °C for 40 min, after a 5 min equilibrium period at the same temperature with agitation in the autosampler. The SPME arrow was then desorbed in splitless mode at 240 °C for 5 min. Compound separation was achieved using a Zebron-Wax capillary column (Phenomenex, CA, USA; 30 m × 0.25 mm ID × 0.25 µm) with helium (99.999% purity, Coregas, NSW, Australia) as the carrier gas, maintaining pressure at 100 kPa. The GC oven temperature was programmed as follows: initially set at 35 °C (held for 2 min), followed by an increase to 250 °C at 6 °C/min, with a final holding time of 22 min. The MS operated in positive electron ionisation (EI) mode at 70 eV, scanning a mass range of *m*/*z* 35 to 220. The ion source and interface temperatures were set to 200 °C and 240 °C, respectively.

A standard mixture of n-alkanes (C_8_–C_20_) was analysed to calculate the retention indices (RIs) of the compounds. Compound identification was achieved by comparing mass spectra (similarity of at least 90%) and RI values (difference of no more than 50) with those in the National Institute of Standards and Technology (NIST, USA) library (version 11). Semi-quantification was based on the peak area of the target ion. Details of tentatively identified compounds, including quantifiers and qualifiers, are provided in Supplementary Materials.

### 2.7. Olfactory Evaluation of Samples

Olfactory evaluation of samples followed the procedure described by [17] and utilised the same semi-trained panel (n = 20, balanced by sex). Sensory panel recruitment and the use of collected data for analysis were approved by the UNSW Human Research Ethics Committee (ethics approval number: 9628). All participants provided their written consent prior to training and evaluation sessions. A total of 4 samples, including prawn (P + DHA + PFM) and salmon (S + DHA + SFM) prototypes in their TS and FTS states, were selected for evaluation. Three grams of each sample were minced and placed into 20 mL glass vials. The four samples were labelled with 3-digit random codes and presented to panellists in randomised order. Odour attributes were evaluated on smart phones using Qualtrics XM platform (Qualtrics, Seattle, WA, USA). Panellists were instructed to sniff the samples and evaluate pre-defined odour attributes using a 100 mm line-scale; overall odour intensity, artificial, cooked crustaceans, cooked salmon, fishy, grilled, herbaceous, marine, meaty, muddy, oxidised oil, vegetable, and smoky odours, as well as using continuous hedonic scales. Odour attributes were defined previously [17], through focus groups and input from experienced flavour chemists. The evaluation was conducted in a single session without replication.

### 2.8. Statistical Analysis

After testing the normality of the data, one-way analysis of variance (ANOVA) followed by Tukey’s post hoc test was performed to compare group means of individual volatile compounds. Multivariate analysis using principal component analysis (PCA) was also conducted. Group means of perceived odour intensities were compared using the Mann–Whitney test. These analyses were conducted using XLSTAT software version 2023.3.1 (1416) (Lumivero, Denver, CO, USA). The PCA biplot and radar charts were visualised using XLSTAT, while the results of TMA pyrolysis and the heatmap of volatile evolution in prawn and salmon prototypes were visualised using GraphPad Prism version 9.5.1 (733) (GraphPad Software, Boston, MA, USA).

## 3. Results and Discussion

### 3.1. Volatile Composition of PB Prawn and Salmon Prototypes

A total of 64 volatile compounds were identified across PB prawn and salmon prototypes with various treatments. These included eight acids, eight alcohols, nineteen aldehydes, four furans, thirteen ketones, ten N-containing compounds, and two S-containing compounds. Significant (*p* < 0.05) differences across samples were observed. A detailed summary comparison of volatiles in each sample compared by one-way ANOVA is provided in Supplementary Materials S1.

**Figure 1:**
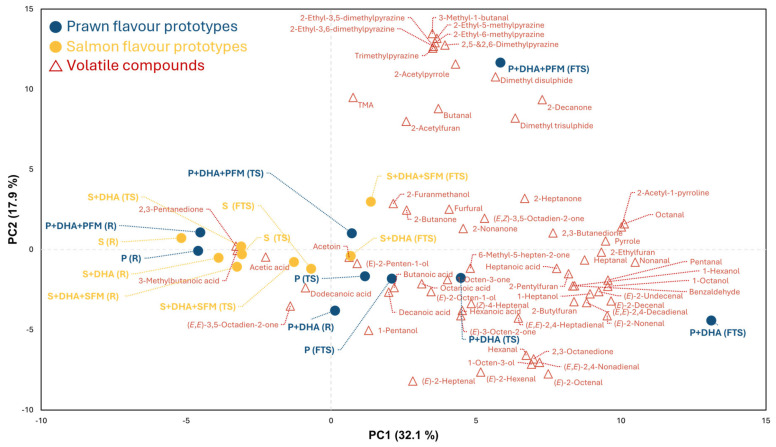
PCA biplot illustrating sample discrimination based on volatile composition. Formulations aiming to mimic prawn and salmon flavours are labelled in blue and yellow, respectively. Thermal treatments are indicated as follows: no thermal treatment applied (R), thermally stabilised (TS), and fried after thermal stabilisation (FTS). Volatile compounds are labelled with red triangles.

To provide a clearer visualisation of the results, PCA was performed, where about 50% of the total variance was explained by the first two principal components (PCs, Figure 1). The positive side of PC1 was associated with high levels of alcohols, aldehydes, ketones, and furans, while the negative side was dominated by 2,3-pentanedione, acetic acid, and 3-methylbutanoic acid. This distribution placed most raw (R) and thermally stabilised (TS) samples for both prawn and salmon prototypes on the negative side of PC1. After subjecting the samples to frying (FTS), the samples shifted to the positive side of PC1, and this was most apparent for the prawn samples P + DHA + PFM and P + DHA. Within each prototype formulation, thermal treatment resulted in a consistent shift toward the positive side of PC1. Additionally, pyrazines and sulphides drove the distinct separation of the P + DHA + PFM (FTS) sample on the positive end of PC2. This trend was also observed for S + DHA + PFM (FTS), but to a lesser extent.

The PCA highlighted the impact of formulation and thermal treatment on volatile profiles, being most strongly influenced by the higher FAAs present in the prawn samples. Typical DHA degradation products were higher in the fried prawn samples, even though they had a much lower lipid and DHA content. Raw PB prawn and salmon prototypes exhibited similar volatile profiles, regardless of the inclusion of DHA or FAA-based flavour mixes. Following thermal stabilisation by steaming, relatively small changes in volatile profiles occurred compared to frying. The vacuum-sealing of prototypes prior to steaming likely limited oxygen exposure and oxidative degradation of PUFAs, especially in salmon prototypes such as S (TS), S + DHA (TS), and S + DHA + SFM (TS), which remained on the negative side of PC1. Frying induced more substantial changes, particularly in PB prawn prototypes. The higher FAA content in prawn formulations likely contributed to the formation of reactive intermediates that accelerated PUFA breakdown, even in low-oxygen conditions [26]. This was reflected in the volatile profiles of P (FTS), P + DHA (FTS), and P + DHA + PFM (FTS). Without PFM, P and P + DHA displayed distinct volatile profiles due to differences in PUFA-derived compounds. The inclusion of PFM resulted in significantly higher levels of pyrazines and sulphide compounds, generated through Strecker degradation and Maillard reactions, creating a profile that was markedly distinct from the other prawn prototypes. A similar trend was observed in S + DHA + SFM (FTS). However, due to the lower FAA content in SFM compared to PFM, the production of pyrazines and sulphide compounds was not as pronounced.

**Figure 2:**
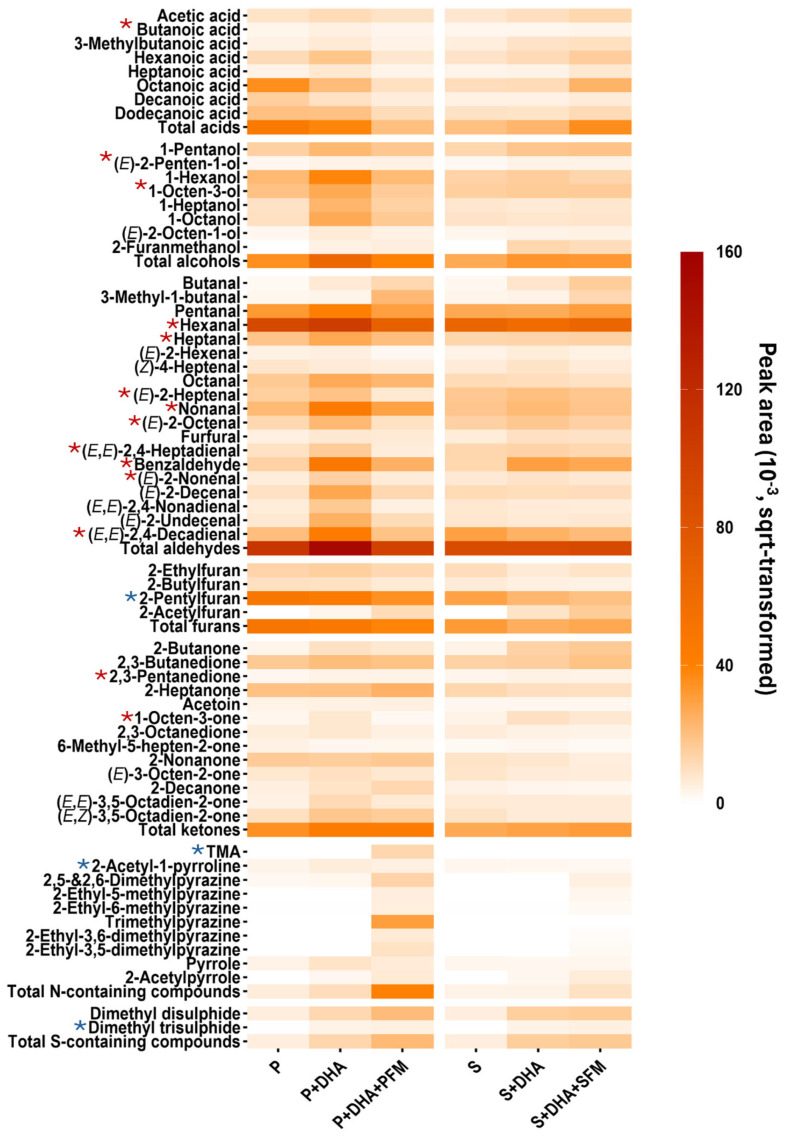
Evolution of volatile compounds in prawn (P, P + DHA, and P + DHA + PFM) and salmon (S, S + DHA, and S + DHA + SFM) prototypes in fried thermally stabilised (FTS) status. Red colours from light to dark indicate concentration gradients from low to high, expressed as 10^−3^ square root-transformed peak area. Odourants identified at particularly high concentrations and classified as key volatiles of prawn and salmon are marked with blue and red “*”, respectively.

The pan-frying process was designed to replicate a typical cooking method for prawn, salmon, and their PB analogues. Consequently, greater emphasis was placed on the flavour evolutionary patterns of FTS prawn and salmon prototypes, which are visualised in Figure 2. The heatmap reveals a higher abundance of volatiles in the PB prawn prototypes compared to the salmon prototypes, with distinct differences in the evolutionary patterns of volatiles across various chemical classes between the two.

For prawn prototypes, the fortification with n-3 PUFAs (P + DHA) and the subsequent inclusion of PFM (P + DHA + PFM) gradually reduced the total acid content, primarily driven by decreases in octanoic, decanoic, and dodecanoic acids. In contrast, an opposite pattern was observed for salmon prototypes, where the acid content increased. The reasons for the decreasing trend in prawn prototypes remain unclear. However, for salmon prototypes, these acids are known to be derived from n-3 PUFAs, and their fortification with DHA likely promotes the production of these acids during frying. This reaction is further enhanced in the presence of FAAs [26].

For alcohols, aldehydes, and furans, the highest abundance was consistently observed in P + DHA amongst the prawn prototypes. Notably, 1-hexanol, 1-hexanal, and 2-pentylfuran were the major contributors to their respective chemical classes in this trend. Compared to P, the addition of DHA in P + DHA likely provided more precursors to produce aldehydes, alcohols, and furans. The reduced levels observed in P + DHA + PFM may suggest a protective mechanism of FAAs against DHA oxidative degradation. This hypothesis is supported by previous studies reporting the antioxidant activities of FAAs [27,28]. Similar patterns were observed in the salmon prototypes, although the trends were less pronounced than in the prawn prototypes. For instance, alcohols and aldehydes were generally most abundant in S + DHA. However, no consistent trend was observed for furans across the salmon prototypes. This complexity was further evident in the ketone compounds. While total ketone contents tended to increase from the PB matrix to the treatment group with added DHA and then to the group fortified with a flavour mix, the specific evolution of individual ketones varied. The highly variable profiles of lipid oxidation-derived compounds in PB prawn and salmon prototypes were closely linked to differences in fat composition, particularly DHA content (Table 2), and the distinct FAA compositions, which likely influenced the fat degradation process, as discussed previously. Furthermore, salmon prototypes (S + DHA and S + DHA + SFM) had a much higher concentration of total fat (20.8%) and DHA (1%) compared to the prawn prototype (1.1% and 0.2%). Theoretically, this would result in a higher production of lipid degradation products, which was not observed. This could be attributed to the higher water content in the prawn formulations, creating a more hydrophilic environment that facilitated the release of non-polar volatiles.

Overall, the addition of the flavour mix enhanced the formation of N- and S-containing volatiles, which are typical products of Strecker degradation of amino acids. This was observed in both PB prawn and salmon prototypes. Additionally, the FAAs (1.8%) and betaine (0.3%) content in the final prawn formulation (P + DHA + PFM) was higher than in the salmon prototype (0.2% and <0.1%, respectively). Consequently, the addition of SFM in S + DHA + SFM resulted in a lower production of N- and S-containing compounds compared to P + DHA + PFM. Pyrazines, including 2-ethyl-5-methylpyrazine, 2-ethyl-6-methylpyrazine, 2-ethyl-3,6-dimethylpyrazine, 2-ethyl-3,5-dimethylpyrazine, dimethyl disulphide, and dimethyl trisulphide, were detected in both S + DHA + SFM and P + DHA + PFM. Notably, trimethylpyrazine was exclusively detected in P + DHA + PFM, and at high levels. This result aligns with the high glycine content in PFM, as glycine is a well-established precursor of trimethylpyrazine [29]. TMA was only detected in P + DHA + PFM, likely resulting from betaine degradation.

### 3.2. Thermal Generation of TMA from Betaine in a Model System

**Figure 3:**
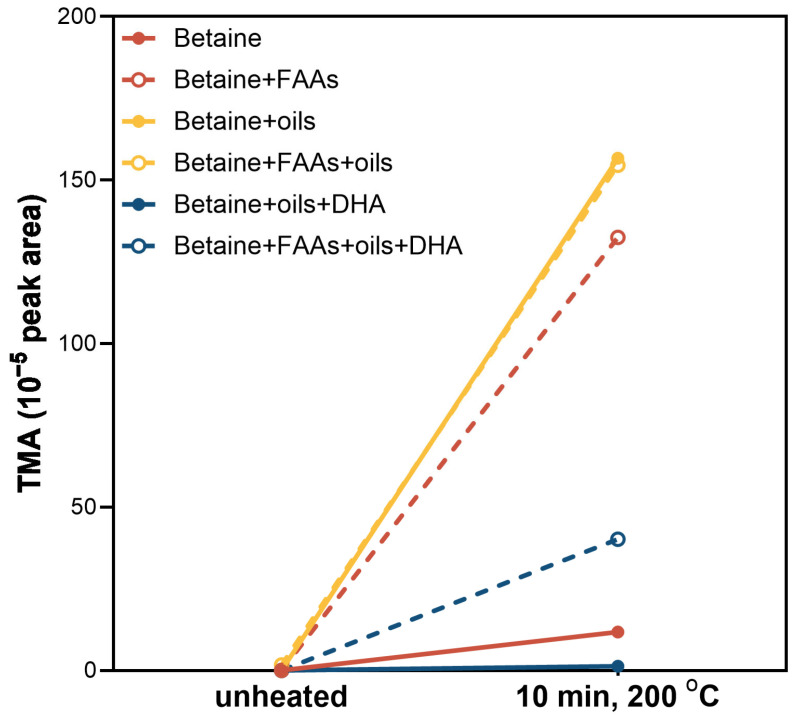
Trimethylamine (TMA) formation through pyrolysis of betaine at 200 °C for 10 min with different ingredients. Treatment groups include betaine alone (betaine, red solid line), betaine with FAA mix (betaine + FAAs, red dashed line), betaine with sunflower and coconut oils (betaine + oils, yellow solid line), betaine with FAA mix and oils (betaine + FAAs + oils, yellow dashed line), betaine with oils and encapsulated algal DHA (betaine + oils + DHA, blue solid line), and betaine with FAA mix, oils, and encapsulated algal DHA (betaine + FAAs + oils + DHA, blue dashed line). TMA content is expressed as 10^−5^ peak area.

While both TMAO and betaine are precursors of TMA in seafood, only betaine is considered suitable for product formulation due to toxicity concerns associated with TMAO. As shown in Figure 3, TMA was not detected by GC–MS in any samples prior to baking. However, after baking at 200 °C for 10 min, TMA was detected in all six treatment groups. Amongst these, TMA production was most pronounced in the betaine + oil and betaine + FAAs + oil groups, followed by betaine + FAAs. The lowest TMA content was observed in betaine + oils + DHA, which was even lower than that of betaine alone. The inclusion of FAAs (betaine + FAAs + oils + DHA) had higher TMA production compared to betaine + oils + DHA.

Suuronen, Pitkänen [30] reported the pyrolysis of betaine using a filament pyrolyser coupled to GC–MS, noting that TMA production only occurred when the temperature exceeded 242 °C. Our results indicated that thermal degradation of betaine to form TMA can occur at 200 °C after 10 min of heating, which is typical of home baking or frying conditions. This suggests that betaine could be utilised to produce TMA after appropriate thermal processing of PBSAs to create a characteristic fish odour. Furthermore, our results indicated that the presence of FAAs enhances betaine pyrolysis, while DHA appears to inhibit the reaction. However, since TMA is primarily formed through the direct reductive cleavage of betaine, the specific roles of FAAs and DHA in this reaction remain unclear and require further investigation.

### 3.3. Presence of Key Prawn and Salmon Odourants in the PB Prototypes

As highlighted in Figure 2, a range of key prawn and salmon odourants, previously identified as abundant and essential in shaping typical seafood characteristics, were detected in the PB prototypes [17]. For instance, key salmon odourants such as butanoic acid (*sweaty*), (*E*)-penten-1-ol (*mushroom*), 1-octen-3-ol (*mushroom*, *green*), hexanal (*green*, *grass*), heptanal (*green*, *floral*), (*E*)-2-heptenal (*grass*, *roasted*), nonanal (*marine*, *plastic*), (*E*)-2-octenal (*nutty*, *fatty*), (*E*,*E*)-2,4-heptadienal (*fatty*, *mushroom*), benzaldehyde (*nutty*, *roasted*), (*E*)-2-nonenal (*earthy*, *green*), (*E*,*E*)-2,4-decadienal (*fatty*, *plastic*), 2,3-pentanedione (*butter*, *caramel*), and 1-octen-3-one (*mushroom*, *green*) were present. These odourants are known to differentiate cooked salmon from other seafood species [8]. Similarly, typical prawn odourants, including 2-pentylfuran (*green*, *grass*), TMA (*fishy*), 2-acetyl-1-pyrroline (*roasted*, *popcorn*), and dimethyl trisulphide (*vegetable*, *sulphur*), were also detected. The presence of these compounds demonstrates the potential of these flavour formulations to impart typical prawn and salmon flavour attributes to the PB matrix.

A subset of key odourants for prawn and salmon was not detected in the samples. Bromophenols, which are abundant in prawns, particularly wild-caught ones, may play a role in imparting a pleasant marine odour. These compounds are accumulated in prawns through their dietary intake of algae. While the DHA used in our formulation was derived from algae, no other algal ingredients were included in the prototypes, which likely explains the absence of bromophenols. Furthermore, Luo, Frank [8] reported the presence of 2,4-dithiapentane (*truffle*) and indole (*faecal*, *musty*) in prawns. The occurrence of these compounds in prawns is likely due to microbial or enzymatic activities that lead to the decomposition of amino acids such as cysteine, methionine, and tryptophan [31,32]. However, such reactions are unlikely to have occurred during the formulation of the prawn prototypes. Furthermore, two key salmon odourants, (*E*,*Z*)-2,6-nonadienal (*cucumber*, *floral*) and (*E*)-3-hexen-1-ol (*fresh*, *green*), reported in our previous study [8], were not detected. Since these compounds are derived from PUFA degradation, their absence suggests that authentic seafood possesses a more complex PUFA profile, which facilitates the production of these characteristic odourants.

### 3.4. Odour Qualities of Prototypes

**Figure 4:**
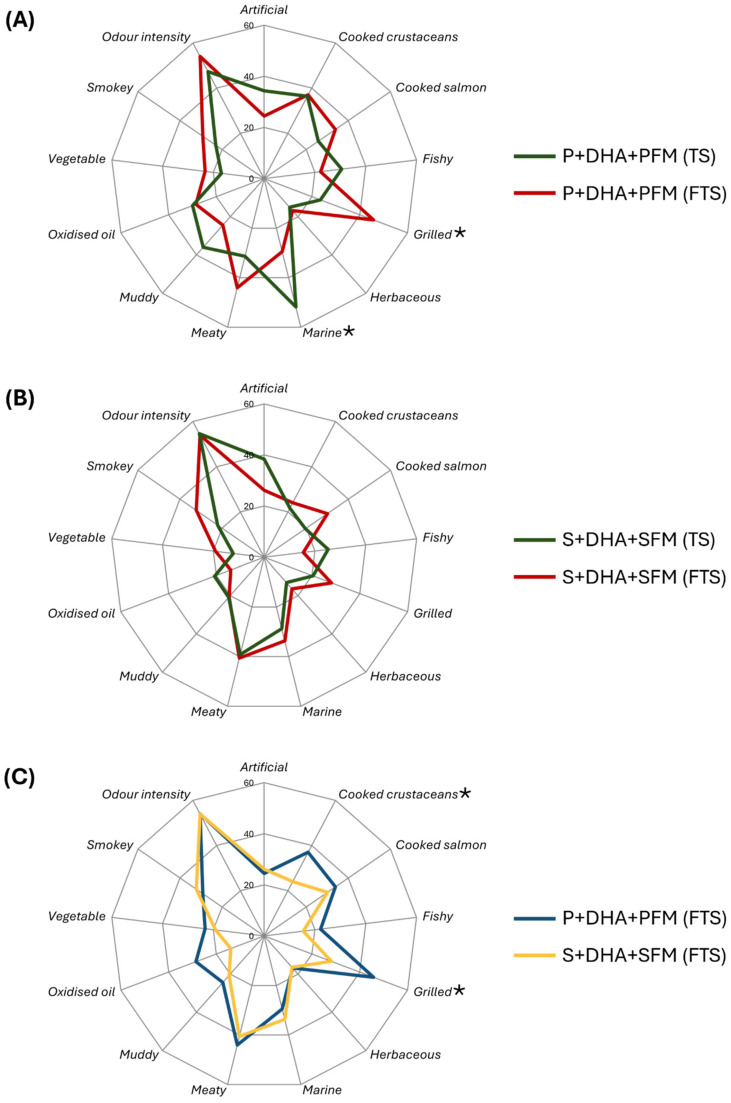
Comparison between odour perception of thermal stablised (TS, green) and fried thermal stablised (FTS, red) P + DHA + PFM prototypes (**A**), TS (green) and FTS (red) S + DHA + SFM prototypes (**B**), and FTS P + DHA + PFM (blue) and FTS S + DHA + SFM prototypes (yellow) (**C**). Significant (*p* < 0.05) differences in perception were tested using the Mann–Whitney test and are marked with “*”.

The odour quality of the final prawn (P + DHA + PFM) and salmon (S + DHA + SFM) prototypes was evaluated through olfactory assessment, with detailed results provided in Supplementary Materials S2. Samples in their TS and FTS states, representing typical shelf and pre-consumption statuses, were selected for the assessment. For the prawn prototypes (Figure 4A), a significantly (*p* < 0.05) stronger grilled note was perceived after frying, aligning with the observed increase in N- and S-containing compounds associated with grilled aromas in the volatile analysis. Interestingly, a significant decrease in the strength of marine odour was also noted after frying. Since the formulation did not include typical marine odour-contributing compounds (e.g., bromophenols) apart from algal DHA, this decrease might be attributed to the odour-masking effect of the grilled notes. For the salmon prototypes (Figure 4B), no statistically significant differences in the assessed odour attributes were observed, which could be explained by the relatively similar volatile profiles of S + DHA + SFM before and after frying (Figure 1). Overall, for both prawn and salmon prototypes, the changes in odour quality before and after frying were minimal. This aligns with previous findings indicating that the volatile profile changes in commercial PB seafood alternatives before and after cooking are minor compared to authentic seafood [17].

When comparing the two final formulations after frying (Figure 4C), most odour attributes showed similar perceived intensities. However, the prawn prototype (P + DHA + PFM) exhibited significantly (*p* < 0.05) stronger cooked crustacean and grilled notes. This suggests that fortifying the formulation with FAAs and betaine can effectively enhance crustacean-like flavour attributes in PB products. On the other hand, a significantly stronger cooked salmon odour was not perceived in the DHA-fortified formulation (S + DHA + SFM). This finding may imply that the characteristic salmon odour profile involves a more complex combination of odourants, beyond those derived solely from algal n-3 PUFAs.

## 4. Conclusions

Commercial PB seafood flavourings, primarily formulated with plant- and yeast-derived ingredients, struggle to replicate authentic seafood flavours, which significantly contributes to the often-disappointing flavour quality of commercial PBSAs. In this study, we examined the feasibility of a flavouring strategy for PB prawn and salmon products. Using natural levels of n-3 PUFAs, FAAs, and betaine as references, different formulations were designed. Volatile profiles of the prototypes were analysed through HS-SPME-GC–MS, and odour quality was evaluated via olfactory assessment.

Our findings reveal significant differences in the volatile compositions of PB prawn and salmon formulations, depending on the combination of DHA, FAAs, and betaine. A range of key prawn and salmon odourants was detected in prototypes fortified with all three flavour precursors. Notably, this study also indicated the potential production of TMA through the pyrolysis of betaine under moderate cooking conditions. The development of cooked crustacean odours in the final prawn prototype after frying was validated by a semi-trained panel, demonstrating the potential of this flavouring strategy in producing PB prawn products with flavour attributes that more closely resemble authentic prawn. While the final salmon prototype contained key salmon odourants, the cooked salmon odour was not pronounced. Perhaps, fortifying the PB matrix with a more robust n-3 PUFA profile that better mimics authentic salmon, for example, including eicosapentaenoic acid, could better enhance its flavour quality.

This flavouring strategy could also be extended to mimic other popular PBSAs, such as PB scallop and squid, whose key flavourants and flavour precursors have been well-studied. Additionally, future research could focus on incorporating other odourants that, although present in trace amounts, play a critical role in shaping characteristic aromas. For instance, a more pronounced marine odour, attributed to bromophenols, could be achieved by incorporating seaweed or algal oil enriched in bromophenols in the formulation.

## Figures and Tables

**Table 1:** PB prawn and salmon formulation targets.

	Prawn	Salmon
**Macronutrients (g/100 g fresh weight) ^1^**		
Protein	17.5	19.3
Saturated fat	0.2	4.7
Unsaturated fat	0.7	15.1
n-3 PUFAs	0.24	2.8
Sodium	0.25	0.03
**FAAs (mg/100 g fresh weight) ^2^**		
Alanine	83.5	57.1
Arginine	420.3	7.4
Aspartic acid	1.9	0.5
Glutamic acid	31.7	17.7
Glycine	660.3	44.1
Histidine	43.9	31.1
Leucine	33.4	4.5
Lysine	38.7	3.9
Methionine	13.8	1.5
Proline	370.8	2.8
Valine	39.5	10.2
Cystine	1.1	0.1
Cysteine	0.5	0.1
Phenylalanine	16.1	4.7
**Other (mg/100 g fresh weight) ^3^**		
Betaine	357.1	0.6

Notes: ^1^ Data were obtained from the Australian Food Composition Database (available at https://www.foodstandards.gov.au/science-data/monitoringnutrients/afcd, accessed on 12 December 2024). ^2^ Values were adapted from the studies of Vázquez-Ortiz, Caire [23] and Zhang, Chen [24] and then converted into salmon targets based on relative ratios determined in our previous study [8]. ^3^ Betaine targets for both prawn and salmon were calculated using literature values from squid [25].

**Table 2:** Formulation and odour quality of prototypes.

	Prawn Prototypes			Salmon Prototypes		
	*P*	*P + D HA*	*P + DHA + PFM*	*S*	*S + DHA*	*S + DHA + SFM*
**Ingredients (g/100 g fresh weight)**						
Faba protein isolate	10.0	10.0	10.0	10.0	10.0	10.0
Encapsulated algal DHA	0	2.4	2.4	0	10	10
Total fat ^1^	0.9	1.1	1.1	19.8	20.8	20.8
- Sunflower oil	0.7	0.7	0.7	15.1	15.1	15.1
- Coconut oil	0.2	0.2	0.2	4.7	4.7	4.7
- DHA (from encapsulated algal DHA)	0	0.2	0.2	0	1	1
Flavour mix	0	0	2.1	0	0	0.2
- FAAs ^2^	0	0	1.8	0	0	0.2
- Betaine ^3^	0	0	0.3	0	0	<0.1
NaCl	0.2	0.2	0.2	0.3	0.3	0.3
Gelling agent	5.0	5.0	5.0	5.0	5.0	5.0
CaCl_2_	1.0	1.0	1.0	1.0	1.0	1.0
MQ water	80.4	80.4	78.3	54.0	54.0	53.7
Flavour mix	0	0	2.1	0	0	0.2
Algal DHA	0	2.4	2.4	0	10	10
Tapioca flour	2.4	0	0	10.0	0	0
**Odour quality ^4^**						
Unheated mixture (R)	very mild beany, neutral	very mild beany, neutral	mild fresh seafood, not unpleasant, savoury	very mild fatty, neutral	fresh fish, mild fatty, not unpleasant	neutral, very mild fatty, fresh fish
Thermal stabilised (TS)	mild oxidised fat, very mild beany	very mild fish oil, not unpleasant, neutral	very mild crustacean, not unpleasant	very mild fatty, beany	fatty, fresh fish, mild, not unpleasant	mild oxidised fish oil, slightly fishy, not unpleasant
Fried thermal stabilised (FTS)	mild beany, fatty	mild fried fish, fatty	cooked crustacean, fried fish	mild salmon, fatty, fired food	fried oily fish, fried salmon	fried oily fish, fried salmon

Notes: ^1^ The total content of sunflower oil, coconut oil, and encapsulated algal DHA (containing no less than 10% DHA) in the prototype. ^2^ The total content of all listed FAAs in Table 1. ^3^ Betaine content listed in Table 1. ^4^ Odour qualities assessed and recorded by two experienced flavour chemists.

## Data Availability

The original contributions presented in the study are included in the article/Supplementary Materials. Further inquiries can be directed to the corresponding author.

## Supplementary Materials

The following supporting information can be downloaded at: https://www.mdpi.com/article/10.3390/foods14050820/s1, Supplementary Materials S1: compound list; Supplementary Materials S2: Olfactory evaluation; Supplementary Table S1: Composition of model reaction mixes used in the betaine pyrolysis trial.
