# Supplementary material for: Forging Prawn and Salmon Flavours with Non-Animal-Based Ingredients
Source: Foods. 2025 Feb 27;14(5):820. doi: 10.3390/foods14050820 (PMC11898523; doi:10.3390/foods14050820)
Supplement: Supplementary file 1 [file foods-14-00820-s001.zip › Supplementary Table.pdf]

Supplementary Table S1. Composition of model reaction mixes used in the betaine pyrolysis trial.

	Model reaction mixes					
	Betaine	*Betaine+FAAs*	*Betaine+oils*	*Betaine+FAAs+oils*	*Betaine+oils+DHA*	*Betaine+FAAs+oils+DHA*
**Ingredients**						
Betaine (g)	0.4	0.4	0.4	0.4	0.4	0.4
FAAs (g)	0	1.8	0	1.8	0	1.8
-Alanine (mg)	0	83.5	0	83.5	0	83.5
-Arginine (mg)	0	420.3	0	420.3	0	420.3
-Aspartic acid (mg)	0	1.9	0	1.9	0	1.9
-Glutamic acid (mg)	0	31.7	0	31.7	0	31.7
-Glycine (mg)	0	660.3	0	660.3	0	660.3
-Histidine (mg)	0	43.9	0	43.9	0	43.9
-Leucine (mg)	0	33.4	0	33.4	0	33.4
-Lysine (mg)	0	38.7	0	38.7	0	38.7
-Methionine (mg)	0	13.8	0	13.8	0	13.8
-Proline (mg)	0	370.8	0	370.8	0	370.8
-Valine (mg)	0	39.5	0	39.5	0	39.5
-Cystine (mg)	0	1.1	0	1.1	0	1.1
-Cysteine (mg)	0	0.5	0	0.5	0	0.5
-Phenylalanine (mg)	0	16.1	0	16.1	0	16.1
Oils (g)	0	0	0.9	0.9	0.9	0.9
-Sunflower oil (g)	0	0	0.7	0.7	0.7	0.7
-Coconut oil (g)	0	0	0.2	0.2	0.2	0.2
Encapsulated algal DHA (g)	0	0	0	0	2.4	2.4
